# Carotid endarterectomy with patch angioplasty versus primary closure in patients with symptomatic and significant stenosis: a systematic review with meta-analyses and trial sequential analysis of randomized clinical trials

**DOI:** 10.1186/s13643-021-01692-8

**Published:** 2021-05-06

**Authors:** Martijn S. Marsman, Jørn Wetterslev, Abdelkarime Kh. Jahrome, Christian Gluud, Frans L. Moll, Frederik Keus, Giel G. Koning

**Affiliations:** 1 Department of Vascular Surgery, Rijnstate Hospital, Wagnerlaan 55, 6815 AD Arnhem, the Netherlands; 2 Copenhagen Trial Unit, Centre for Clinical Intervention Research, The Capital Region of Denmark, Rigshospitalet, Copenhagen University Hospital, Copenhagen, Denmark; 3 Department of Vascular Surgery, Medical Center Leeuwarden, Leeuwarden, the Netherlands; 4 Institute of Regional Health Research, Faculty of Health Sciences, University of Southern Denmark, Odense, Denmark; 5 Department of Vascular Surgery, University Medical Center Utrecht, Utrecht, the Netherlands; 6 Department of Critical Care, University of Groningen, University Medical Center Groningen, Groningen, the Netherlands; 7 Department of Vascular Surgery, ZGT, Hospital Group Twente, Almelo/Hengelo, the Netherlands

**Keywords:** Carotid stenosis, Carotid endarterectomy, Patch, Systematic review, Primary closure, Trial sequential analysis, GRADE

## Abstract

**Background:**

Patch angioplasty in conventional carotid endarterectomy is suggested to reduce the risk of restenosis and recurrent ipsilateral stroke compared with primary closure. A systematic review of randomized clinical trials is needed to compare outcomes (benefits and harms) of both techniques.

**Methods:**

Searches (CENTRAL, PubMed/MEDLINE, EMBASE, and other databases) were last updated 3rd of January 2021. We included randomized clinical trials comparing carotid endarterectomy with patch angioplasty versus primary closure of the arterial wall in patients with a symptomatic and significant (> 50%) carotid stenosis. Primary outcomes are defined as all-cause mortality and serious adverse events.

**Results:**

We included 12 randomized clinical trials including 2187 participants who underwent 2335 operations for carotid stenosis comparing carotid endarterectomy with patch closure (1280 operations) versus carotid endarterectomy with primary closure (1055 operations). Meta-analysis comparing carotid endarterectomy with patch angioplasty versus carotid endarterectomy with primary closure may potentially decrease the number of patients with all-cause mortality (RR 0.53; 95% CI 0.26 to 1.08; *p* = 0.08, best-case scenario for patch), serious adverse events (RR 0.73; 95% CI 0.56 to 0.96; *p* = 0.02, best-case scenario for patch), and the number of restenosis (RR 0.41; 95% CI 0.23 to 0.71; *p* < 0.01). Trial sequential analysis demonstrated that the required information sizes were far from being reached for these patient-important outcomes. All the patient-relevant outcomes were at low certainty of evidence according to The Grading of Recommendations Assessment, Development, and Evaluation.

**Conclusions:**

This systematic review showed no conclusive evidence of a difference between carotid endarterectomy with patch angioplasty versus primary closure of the arterial wall on all-cause mortality, < 30 days mortality, < 30 days stroke, or any other serious adverse events. These conclusions are based on data from 15 to 35 years ago, obtained in trials with very low certainty according to GRADE, and should be interpreted cautiously. Therefore, we suggest conducting new randomized clinical trials patch angioplasty versus primary closure in carotid endarterectomy in symptomatic patients with an internal carotid artery stenosis of 50% or more. Such trials ought to be designed according to the Standard Protocol Items: Recommendations for Interventional Trials statement (Chan et al., Ann Intern Med 1:200–7, 2013) and reported according to the Consolidated Standards of Reporting Trials statement (Schulz et al., 7, 2010). Until conclusive evidence is obtained, the standard of care according to guidelines should not be abandoned.

**Systematic review registration:**

PROSPERO CRD42014013416. Review protocol publication 2019 DOI: 10.1136/bmjopen-2018-026419.

**Supplementary Information:**

The online version contains supplementary material available at 10.1186/s13643-021-01692-8.

## Background

Carotid artery stenosis occurs due to atherosclerosis and was described to be a pathologic substrate for ischemic diseases of the ipsilateral brain and eye by C. Miller Fisher in 1951 [1]. The preventive management of asymptomatic carotid artery stenosis is well known, including antiplatelet therapy, statins, antihypertensive medication, diabetic control, and lifestyle modifications [2–4]. Carotid endarterectomy (CEA) is the preferred guideline treatment for patients with symptomatic and > 50% stenosis of the internal carotid artery [5], based primarily on the European Carotid Surgery Trial (ECST) and the North American Symptomatic Carotid Endarterectomy Trial (NASCET) [6–8].

**Fig. 1:**
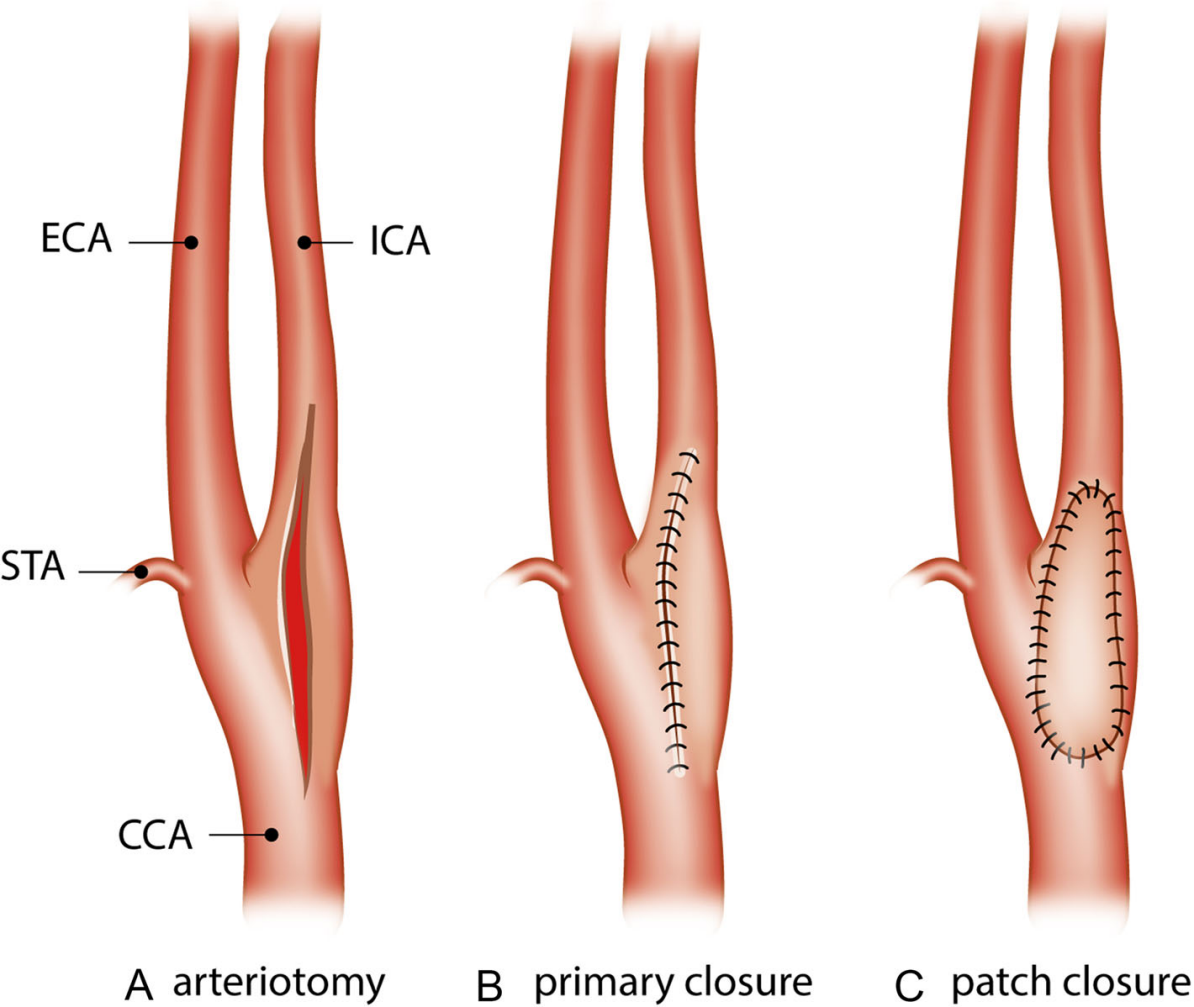
Closure of carotid artery. CCA: common carotid artery, STA: superior thyroid artery, ECA: external carotid artery, ICA: internal carotid artery. **a** Longitudinal arteriotomy. **b** Primary closure of longitudinal arteriotomy. **c** Closure of longitudinal arteriotomy with patch angioplasty

Restenosis after CEA occurs in 6 to 36% of patients during follow-up of 12 months or more [9–13]. Two operative techniques are well known in literature: the eversion technique and the traditional (or conventional) carotid endarterectomy (CEA) using a longitudinal arteriotomy. Closure of the arterial wall in CEA can be achieved by either patch angioplasty or direct suturing of the arterial wall (Fig. 1a–c) [14]. Use of patch angioplasty in CEA is suggested to reduce both the risks of restenosis and recurrent ipsilateral stroke [15].

Guidelines of both the European Society of Vascular Surgery (ESVS) and the Dutch Society for Vascular Surgery (NVvV) consider CEA with patch angioplasty the recommended technique in patients with a severe (> 50%) and symptomatic stenosis. For asymptomatic patients with an “average surgical risk” and a 60 to 99% stenosis, CEA should be considered in the presence of one or more imaging characteristics that may be associated with an increased risk of late ipsilateral stroke [8, 16, 17]. A meta-analysis of ten randomized clinical trials (RCTs) including 2157 procedures in 1967 patients compared CEA with patch angioplasty versus CEA with primary closure of the arterial wall and concluded that the former may reduce the risks of restenosis, perioperative arterial occlusion, and ipsilateral stroke [15]. However, the observed differences in intervention effects may or may not be affected by confounding factors and/or differential use of co-interventions, such as the use of perioperative transcranial Doppler monitoring, perioperative carotid pressure measurement, electroencephalographic monitoring, selected use of shunting, regional anesthesia, and variations in materials used for patching [18–25]. Furthermore, patch angioplasty is not fully accepted by surgeons and several large observational studies failed to find improved outcomes in comparison to primary closure [26–29].

To determine which technique offers superiority of outcomes (e.g., less postoperative (< 30 days) mortality and postoperative (< 30 days) stroke) for patients with a symptomatic stenosis of the internal carotid artery > 50%, it is important that all available evidence is evaluated in a systematic review in accordance with the Cochrane Handbook for Systematic Reviews of Interventions [30, 31]. Therefore, an updated systematic review with meta-analyses is needed. To confirm or reject the meta-analysis results, we used trial sequential analysis (TSA) [32]. This review evaluates the current available evidence and assesses if this evidence is solid enough to base our current practice on it, while taking into consideration the changes in recent years (e.g., best medical treatment, use of ultrasound, centralization, and patch material).

### Why is this systematic review needed?

To date, all reviews suggest patch angioplasty to be superior to primary closure, but it has not been unequivocally proven [33, 34]. Solid recommendations are needed for future research to guide clinical practice.

### Strengths and limitations of this study

- This review was conducted according to the published protocol following the recommendations of Cochrane and reported according to the Preferred Reporting Items for Systematic Reviews and Meta-Analyses (PRISMA statement).
- Trial sequential analysis and Grading of Recommendations Assessment, Development and Evaluation (GRADE) assessments of randomized clinical trials are conducted.
- This review benefits from a comprehensive search strategy, designed to retrieve a broad spectrum of relevant randomized clinical trials for the research question.
- To avoid confounding, **one** technique was compared to **one** other operative technique.

## Methods

This review was conducted according to the published protocol in BMJ Open [32] (see Supplementary file 4). The protocol was registered in PROSPERO in 2014, and this protocol was updated in October 2018 (CRD42014013416) following the recommendations of the “Cochrane Handbook for Systematic Reviews of Interventions” [30] and was reported according to the Preferred Reporting Items for Systematic Reviews and Meta-Analyses (PRISMA) statement [35]. The overall search was last updated on the 3rd of January 2021.

### Studies

Only RCTs comparing CEA with patch angioplasty (regardless of used patch materials) versus CEA with primary closure of the arterial wall were included.

### Patients

According to the current guidelines, patients with a symptomatic stenosis > 50% (measured by ultrasound duplex, computed tomographic angiography, or magnetic resonance angiography) of the internal carotid artery were considered [6–8].

### Experimental intervention

The experimental intervention was CEA with patch angioplasty regardless of the type of patch material used (Fig. 1c) [14].

### Control intervention

The control intervention (Fig. 1b) was traditional CEA (with longitudinal arteriotomy, Fig. 1a) with primary closure of the arterial wall [14]. RCTs that compared the eversion technique versus carotid endarterectomy with patch angioplasty were excluded [36]. The reason for excluding the eversion technique is because it is a completely different technique. When performing the eversion technique, the internal carotid artery (ICA) is cut loose from the common carotid artery (CCA). After removing the atherosclerotic plaque, the ICA will be reinserted at the CCA. Eversion technique is a non-patch technique.

In this review, patch closure (experimental group) is compared with primary closure (control group). Both techniques start with an arteriotomy of the internal carotid artery. After removing the plaque, the ICA can be closed directly (with sutures); this is called primary closure. The ICA can also be closed with a patch (which is sutured); this is called patch closure (Fig. 1).

### Outcomes

The outcome measures were graded from the patients’ perspective (GRADE Working Group 2008, Fig. 2) [37]. The authors graded these aspects from the patients’ perspective. This assumption will be checked in a patient focus group discussion. The number of patients with one or more complications was assessed rather than the number of events, depending on the availability of data (to reduce the risk for double counting).

**Fig. 2:**
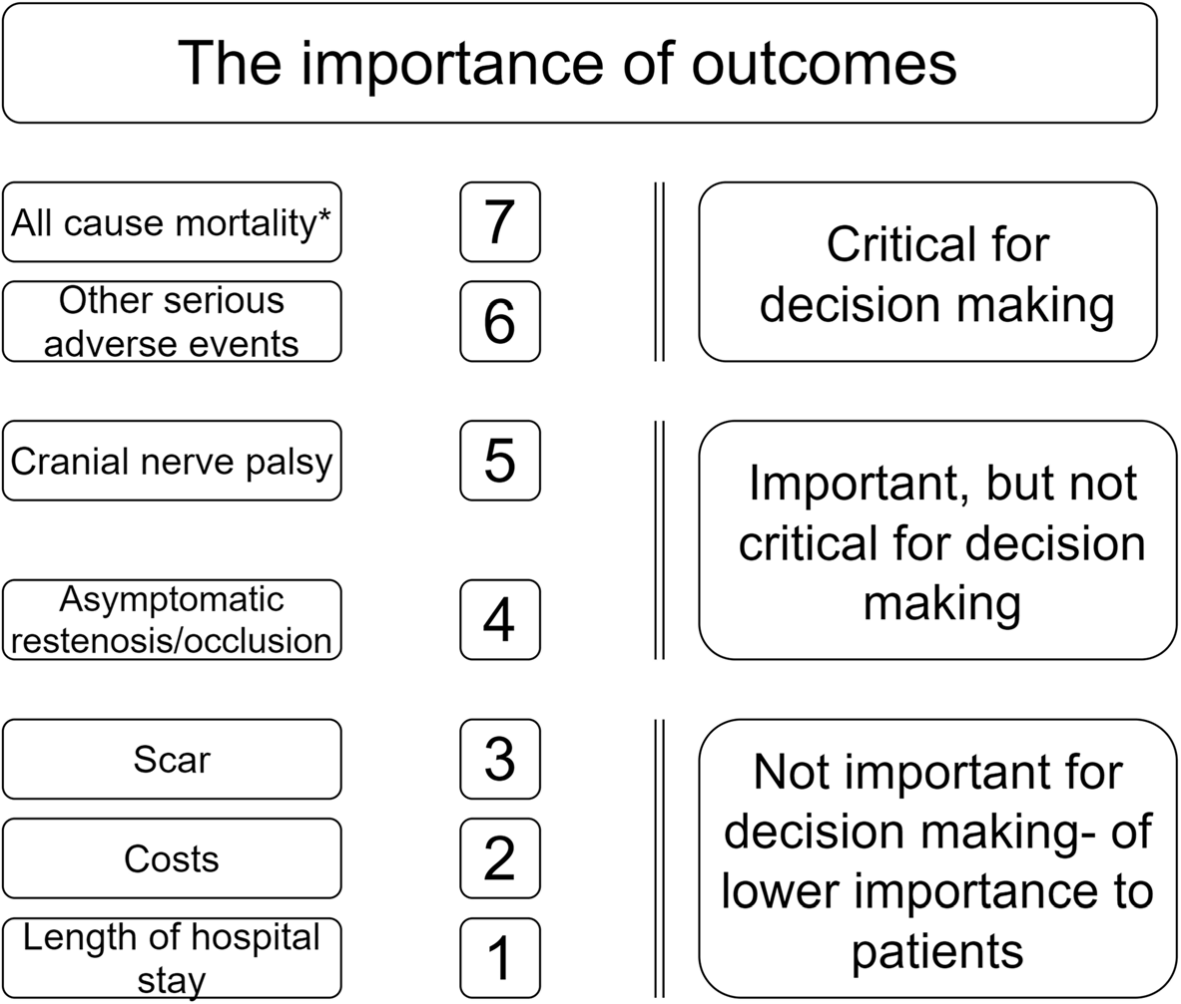
Hierarchy of outcomes from patients’ perspective undergoing carotid endarterectomy for symptomatic carotid stenosis (GRADE 2008). *At maximum follow-up. Serious adverse events such as neck hematoma and cranial nerve injury. Other serious adverse events include stroke < 30 days

#### Primary outcomes

- All-cause mortality
- Proportion of participants with one or more serious adverse events: any untoward medical occurrence that results in death, is life-threatening, requires hospitalization or prolongation of existing hospitalization, results in persistent or significant disability or incapacity [38]
- Health-related quality of life: any scale used by trialists to assess the participants’ reporting of their quality of life (or health status)

#### Secondary outcomes

- Symptomatic or asymptomatic arterial occlusion or restenosis (50 to 99%)
- Proportion of participants with one or more non-serious adverse events: any untoward medical occurrence in a participant who did not meet the above criteria for a serious adverse event was defined as a non-serious adverse event [38]

#### Exploratory outcomes

- Separately reported serious adverse events
- Separately reported non-serious adverse events
- Postoperative stroke rate (< 30 days)

### Search strategy

**Fig. 3:**
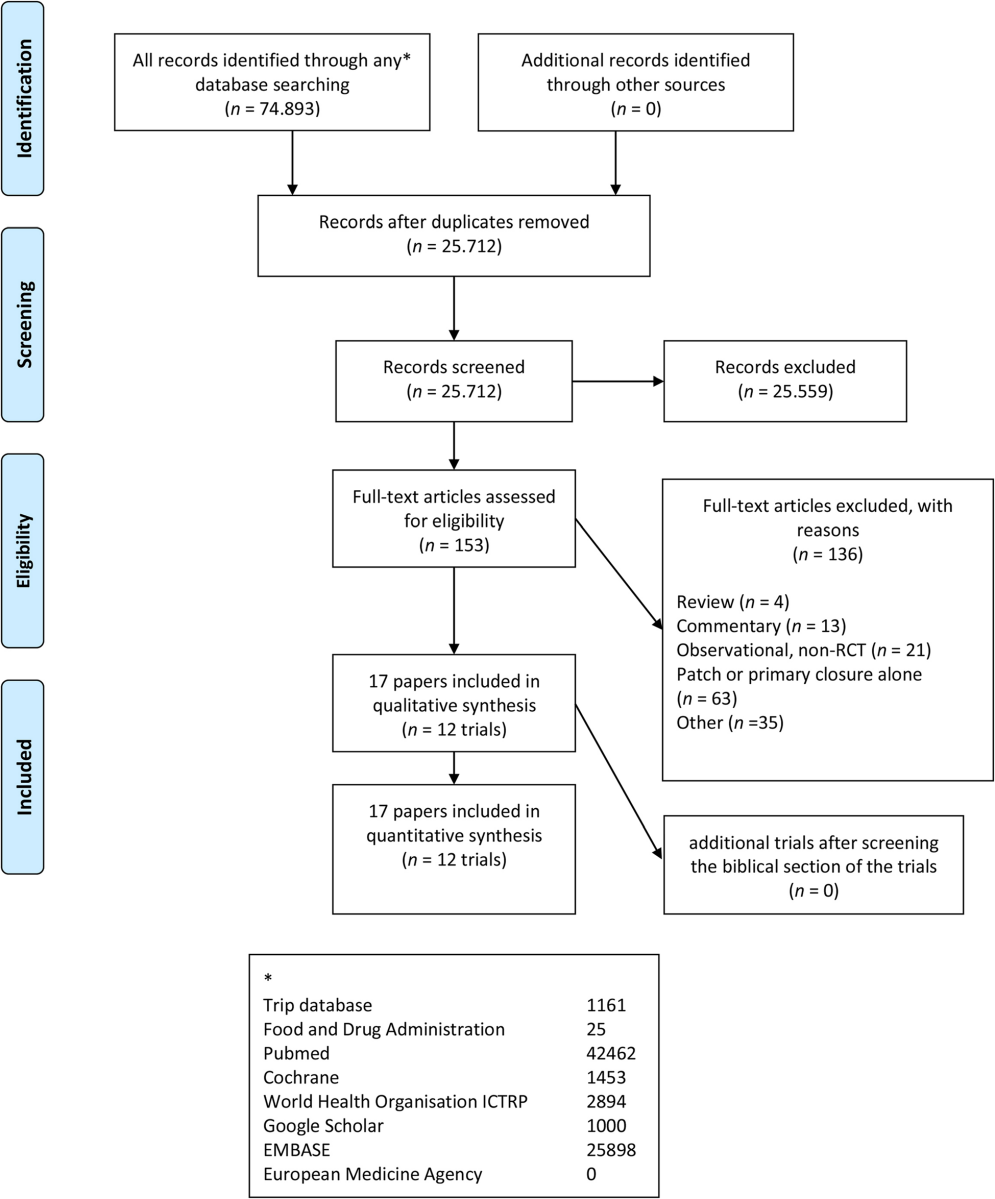
Flow diagram summarizing the search process and results of each phase of the systematic review. doi:10.1371/journal.pmed1000097

The Cochrane Central Register of Controlled Trials (CENTRAL) in The Cochrane Library, PubMed/MEDLINE, EMBASE, and other databases were searched (other databases are summed up in Fig. 3). References of the identified trials were searched to identify any further relevant RCTs. We also searched online trial registries [32]. The search strategy is added as supplementary file 1.

### Data collection

Two authors (MSM and GGK) independently performed screening and selected the trials for inclusion. Excluded trials and studies were listed with their reasons for exclusion. When disagreements occurred, a third author (JW and/or CG) was approached to reconcile. If there was any unclear or missing data, the corresponding authors of the individual trials were contacted at least twice.

### Risk of bias assessment

Two authors (MSM and GGK) assessed the risks of bias, without masking for trial names, according to the Cochrane Handbook for Systematic Reviews of Interventions as described in the published protocol [30, 32].

### Differences between the protocol and this paper

We interchanged the control and experimental interventions after careful considerations within the author group. In our protocol, primary closure is stated as the experimental intervention and patch closure as the control intervention. We recognized the need for this change as the primary closure technique was first known, and patch closure technique was developed later. Critical reviewing from non-involved vascular experts made us adapt the description of the outcomes. These added outcomes (< 30-day stroke and < 30-day mortality rate) are considered critical for decision making from the patients’ perspective and were therefore added as exploratory outcomes to this paper. All participants in the trials were investigated, and all patients with one or more strokes were scored in the outcome serious adverse events. Not all trials reported a number of patients with one or more complications or a number of patients in each intervention group; therefore, meta-analysis is done at the number of surgeries instead of number of patients. In this scenario, the number of surgeries was counted, hereby underestimating the proportion of having a complication. An addendum was made in this paper for the legend of Fig. 2 compared to the protocol. In the protocol, it may look like stroke was considered not to be critical for decision making from patients’ perspective; nevertheless, it is critical for decision making. Also the degree of stenosis at a threshold of 70% for symptomatic patients was changed into 50% according to the latest expert consensus and common daily practice. The overall search was repeated on the 3rd of January 2021. The degree of stenosis of each patient was not explicitly specified in each trial. This lack of data and the very low response from the corresponding authors after several requests resulted in an undefined mix of symptomatic and asymptomatic patients. Additional screening using the Risk of Bias 2 [39] did not reveal any new insights, and all trials were considered at high risk of bias.

### Statistical methods and trial sequential analyses

Meta-analyses were performed according to the Cochrane Handbook for Systematic Reviews of Interventions [30]. The software package Review Manager (RevMan) version 5.3 was used [40]. Significance levels were adjusted due to multiplicity of several outcomes. The results of each outcome were determinative for the use of the intervention and require an adjusted statistical significance level (threshold). An alpha of 0.05/ (1 + 3)/2 = 0.025 was planned to use for the primary outcomes to keep the family wise error rate (FWER) < 0.05. Because health-related quality of life was not analyzed, we chose to adjust maximal type I error for each analyzed outcome to 0.033% to preserve a FWER of 0.05. For the secondary outcomes, the alpha was also 0.033 [41, 42]. For exploratory outcomes, we considered a *p* value < 0.05 as significant, because we viewed these outcomes as only hypothesis-generating outcomes. For dichotomous variables, the risk ratio with 95% confidence interval (CI) was calculated. For continuous variables, the mean difference (MD) or the standardized mean difference with 95% CI were calculated.

Meta-analyses may result in type-I errors and type-II errors due to an increased risk of random error when sparse data are analyzed and due to repeated significance testing when a cumulative meta-analysis is updated with new trials [43, 44]. To assess the risk of type-I and type-II errors, TSA was used. Detailed TSA description has been published in the protocol [32, 43–45].

A random-effects model and a fixed-effect model were used for meta-analysis in the presence of two or more trials included under the outcomes. In case of discrepancy between the two models, both results were reported. Considering the anticipated abundant clinical heterogeneity, the random-effects model was emphasized except if one or two trials dominated the available evidence. The assumptions behind the two models are different. However, we seldom know which assumptions are correct in each specific case. We chose to present the random-effects model to reflect the weighted average between the results from different populations/trial methods and this average may not apply to all situations.

### Best-case scenario and worst-case scenario

Some of the included trials did not specify in which group an event occurred. Worst-case/best-case scenarios for patch angioplasty were made for all-cause mortality, < 30 days mortality, > 30 days mortality, serious adverse events, and < 30 days stroke.

### GRADE

Summary of findings (SOF) tables (supplementary file 2) were produced. A SOF table for best-case scenario for patch and a worst-case scenario for patch was made. Reasons for downgrading the quality of the available evidence are as follows: risk of bias evaluation of the included bias domains, publication bias, heterogeneity, imprecision, and indirectness (e.g., length of stay is a surrogate outcome measure) [46–48]. We compared the imprecision assessed according to GRADE with that of TSA [49]. No differences were found, and all evidence is graded at very low certainty.

### Patient and public involvement

Patients and/or public were not involved in this study.

## Results

### Study selection

The search resulted in 74,893 hits (Fig. 3). In each step of the selection, the publication was included in any case of doubt. Double publications of trial results were considered as one trial. Based on titles and abstracts, 74,740 publications could be excluded. A total of 153 publications remained for full text evaluation from which 136 were excluded based on protocol criteria. Finally, 17 publications [50–62] describing 12 RCTs were included, published in the period 1986 to2006 [50–54, 59, 61, 62]. Additional data of each trial was requested by contacting the authors repeatedly if needed. None of the included trials used a quasi-randomized design. An updated search on the 3rd of January 2021 showed no new randomized clinical trials on this specific topic.

### Patient characteristics and trial designs

Overall, the 12 included trials randomized 2187 patients and performed 2335 operations for carotid stenosis between CEA with patch closure (1280 operations) versus CEA with primary closure (1055 operations). All 12 trials used similar inclusion criteria, baseline characteristics of the populations were comparable, and all patients undergoing a CEA were included. Concerning the grade of carotid stenosis, the trials reported inconsistently. The specification of exclusion criteria was more clearly reported and included concomitant surgery such as coronary arterial bypass grafting, previous carotid surgery, and small diameter of the internal carotid artery (ICA) (< 4 mm), and abnormal anatomy of the ICA varied and were sometimes not described [61]. Patient characteristics were not extensively described, but no imbalances in age or sex were found (Table S1 and Table 1). The number of patients and procedures differed because some patients were operated on both carotid arteries, sometimes with different techniques on each side. Eight trials used a two-armed parallel group design (patch closure versus primary closure of the arterial wall) and four trials used a three-armed design (2 types of patch material versus primary closure) [51, 53, 62].

**Table 1:** Perioperative characteristics of randomized CEA patients with patch angioplasty versus CEA patients with primary closure of all included trials

Author and year	Anesthesia	TCD	Pressure assessment	Shunt	Asymptomatic
Pratesi 1986 [63]	U	U	U	U	10/100 operations
Vleeschauwer 1987 [50]	U	U	U	Used when indicated	U
Eikelboom 1988 [64]	General	U	U	Used when indicated	23/129 operations
Clagett, 1989 [55]	General	Standardized	U	Always	36/152 operations
Lord 1989 [51]	U	U	Routine	Used when indicated	U
Ranaboldo 1993 [59]	U	U	U	Used when indicated	17/213 operations
Katz 1994 [52]	General	Standardized	U	Standardized	38/100 operations
De Letter 1993 [65]	U	U	U	U	U
Myers 1994 [53]	General	Standardized	U	Standardized	40/163 operations
Aburahma 1996 [62]	General	Standardized	U	Standardized	133/399 operations
Mannheim 2005 [54]	Plexus (9 patients general)	U	Routine	Used when indicated	217/422 operations
Al-Rawi 2006 [61]	General	Standardized	U	Used when indicated	31/328 operations

*TCD* Transcranial Doppler, *U* Unknown, *V* Vein patch, *PTFE* Polytetrafluoroethylene patch, *OV* Obligatory vein patch, *SV* Saphenous vein patch, *JV* Jugular vein patch

### Surgical interventions

Most trials gave a description of the surgical technique. CEA with patch angioplasty versus CEA with primary closure of the arterial wall were performed as described in line with the protocol [32].

### Risk of bias

**Fig. 4:**
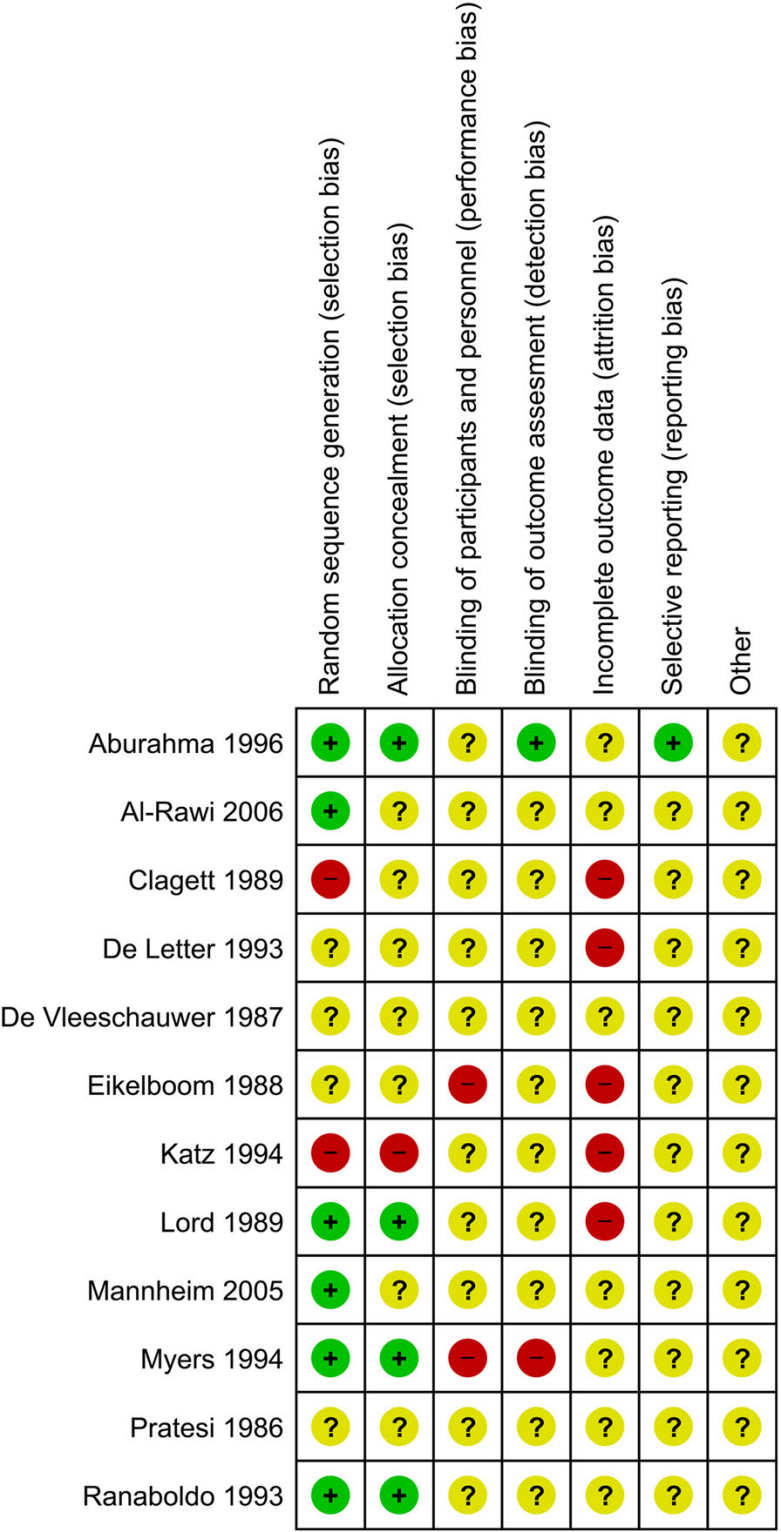
Risk of bias summary of all included trials, the eight criteria on the X-axis. Name of first author and year of trial on *Y*-axis. + = adequate. − = inadequate. question mark (?) = unclear

We assessed the risk of bias of the included trials (Fig. 4). Many bias risk components were unclear. None of the trials used any form of blinding, especially regarding outcome assessment. In 12 trials, one or more out of seven bias components were scored as unclear or at high risk of bias. Therefore, all trials were classified at high risk of bias. Categorization of systematic error (bias) of these clinical intervention studies lead to the level of evidence of each trial of 1d at best [31].

All the available evidence was scored at very low certainty according to GRADE (supplementary file 2).

### Primary outcomes

#### All-cause mortality

**Fig. 5:**
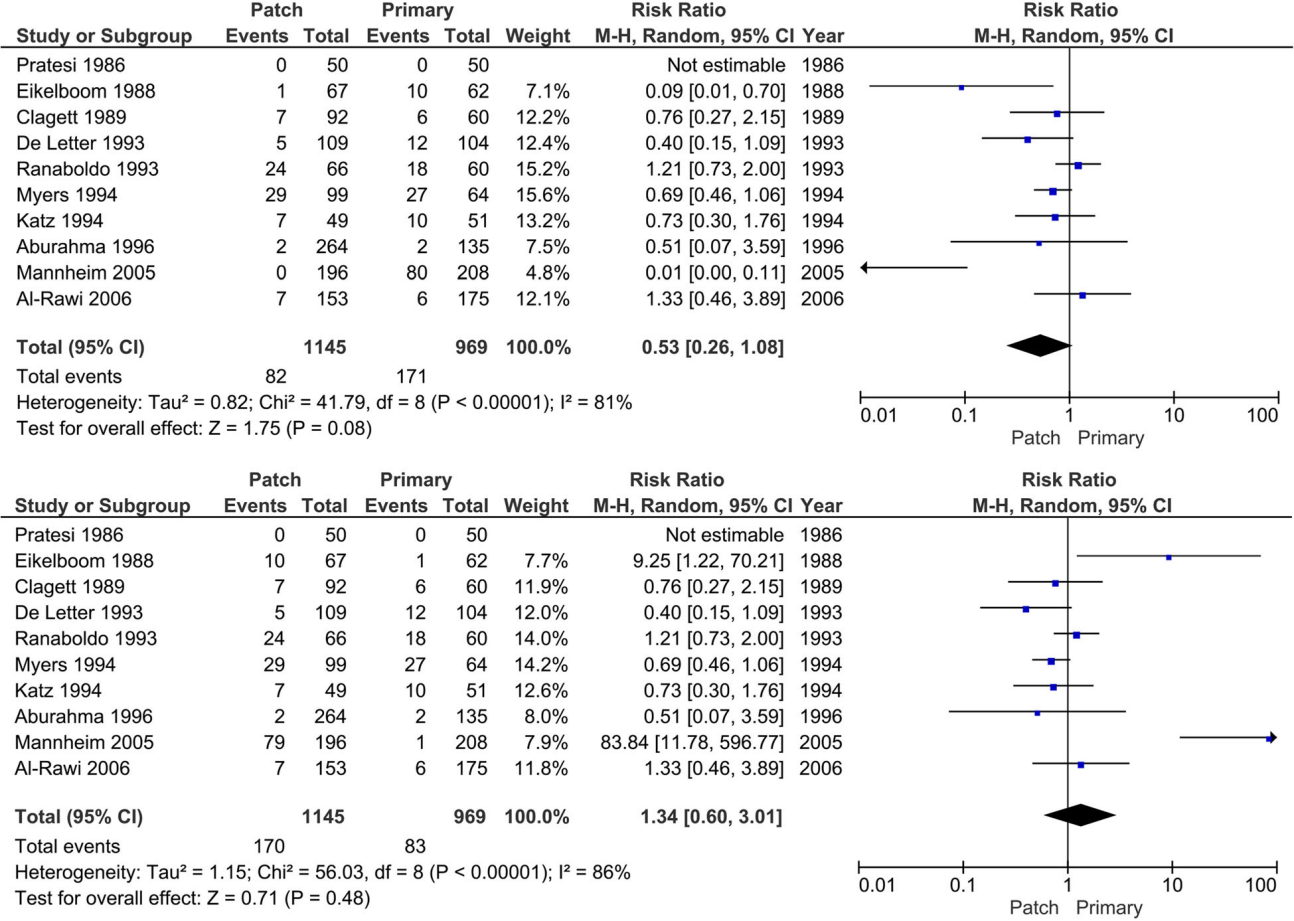
Forest plot on all-cause mortality (random-effect model). **a** Best-case scenario patch angioplasty. **b** Worst-case scenario patch angioplasty

Ten trials reported on all-cause mortality. A total of 1145 operations in the patch angioplasty group and 969 in the primary closure group were reported. In total, 82 patients in the best-case scenario or 171 patients in the worst-case scenario for patch closure (7.2 to 14.8%) died compared with the primary closure group in which 83 patients (worst-case scenario patch) or 171 (best-case scenario patch) (7 to 8.1%) died in 969 operations (Fig. 5). Best-case scenario patch is defined as all the events happened in the primary closure group. Worst-case scenario patch is defined as all the events happened in the patch closure group. In meta-analysis, heterogeneity was present (*I*^2^ 81 to 86%; *p* = < 0.01), and the random-effects model did not show statistical significant differences between the patch angioplasty group versus the primary closure group (RR 0.53; 95% CI 0.26 to 1.08; *p* = 0.08) with very low certainty of evidence (CoE) according to GRADE in the best-case scenario for patch angioplasty. In the worst-case scenario for patch angioplasty, also no significant difference was found (RR 1.34; 95% CI 0.60 to 3.01; *p* = 0.48) The TSA-adjusted CI (or the trial sequential monitoring boundaries) could not be calculated due to too small information fraction (actual information size)/ (required information size), as when this ratio is < 1–2% the TSA program is not able to calculate CI and boundaries. Accordingly, we would have downgraded imprecision at least 1 level with TSA, this was already at the lowest level according to GRADE.

The trial of Eikelboom [64] described nine patients who died, but did not describe to which group they belonged (patch or primary closure). The trial of Mannheim [54] described 79 deaths, also without specifying in which groups. Worst-case/best-case scenario for patch angioplasty was made. In the trial of Pratesi [63], there were zero deaths at maximum follow-up in both arms, patch angioplasty and primary closure. The RevMan program can correct for the zero events in one arm of the trial. When there are zero events in both arms, Revman cannot correct for these zero-zero events. An additional (empirical) analysis was done in the TSA program to correct for these zero-zero events. No differences were found when compared to the Revman analysis. This TSA analysis was also done for mortality < 30 days after surgery (Pratesi, Clagett, Myers, Katz, Al-Rawi [52, 53, 55, 61, 63] had zero-zero counts) and mortality > 30 days after surgery (Pratesi and Aburahma [60, 63] had zero-zero counts). No differences were found when compared to the Revman analysis.

#### Mortality < 30 days after surgery (procedure related)

**Fig. 6:**
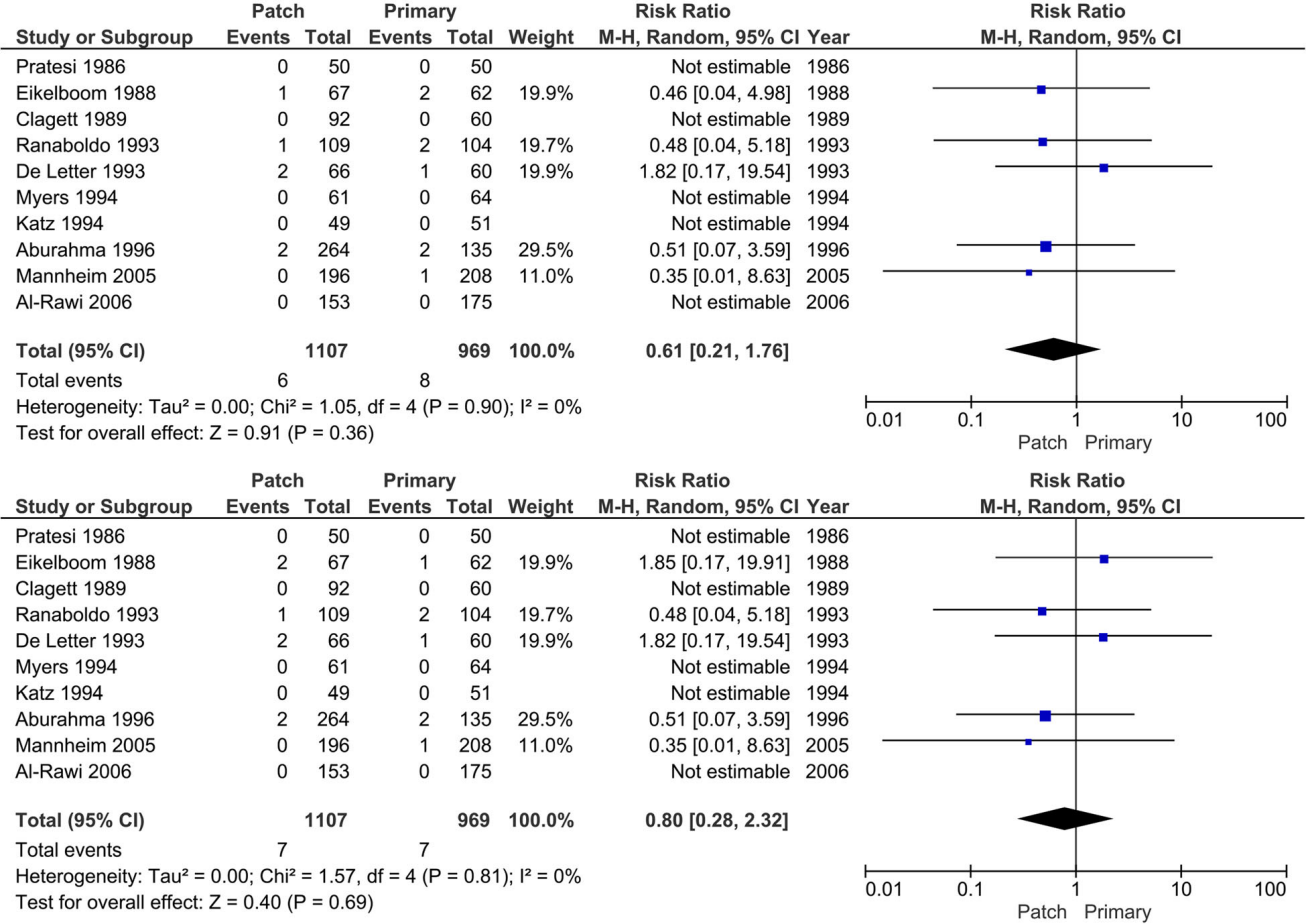
Forest plot on < 30 days (procedure related) mortality (random-effects model). **a** Best-case scenario patch angioplasty. **b** Worst-case scenario patch angioplasty

Six (best scenario for patch) or 7 (worst scenario for patch) patients are described (0.5 to 0.6%) in 1107 operations in the patch angioplasty group compared with 7 (worst-case scenario for patch) or 8 patients (best-case scenario for patch) (0.7 to 0.8%) in 969 operations in the primary closure group who died within 30 days after surgery (Fig. 6). In the patch angioplasty group, 1 patient died due to myocardial infarction, 1 due to respiratory arrest, and 1 from cardiopulmonary arrest. In the primary group, four patients died due to myocardial infarction. In meta-analysis, low heterogeneity was present in both scenarios (*I*^2^ 0%; *p* = 0.81 or *p* = 0.90), and the random-effects model showed no statistically significant differences between the patch angioplasty and primary closure group (RR 0.61; 95% CI 0.21 to 1.76; *p* = 0.36 best-case) and (RR 0.80; 95% CI 0.28 to 2.32; *p* = 0.69 worst-case) with very low CoE. The trial of Eikelboom [64] described one patient who died, but they did not describe in which group. A best-case and worst-case scenario for patch angioplasty was made.

#### Mortality > 30 days (after surgery, non-procedure related)

**Fig. 7:**
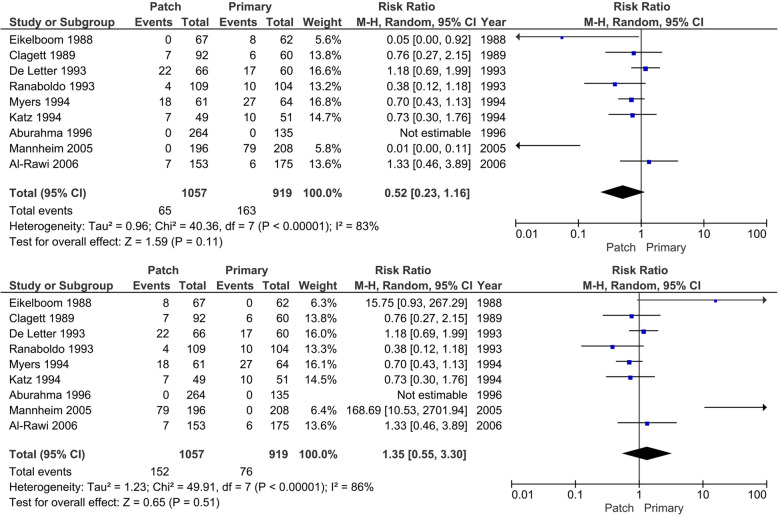
Forest plot on > 30 days mortality (random-effects model). **a** Best-case scenario patch angioplasty. **b** Worst-case scenario patch angioplasty

Sixty-five (best-case scenario patch) or 152 (worst-case scenario patch) patients (6.1 to 14.4%) in 1057 operations in the patch angioplasty group are reported who died > 30 days after surgery for various reasons such as cardiac disease, cancer, and unknown/other (Fig. 7). Compared with the primary closure group in which 76 (worst-case scenario patch) or 163 (best-case scenario patch) patients (8.3 to 15.4%) in 919 operations died > 30 days after surgery. In meta-analysis, heterogeneity was present (*I*^2^ 83 and 86%; *p* = < 0.01), and the random-effects model did not show statistical significant differences between the patch angioplasty and the primary closure group (RR 0.52; 95% CI 0.23 to 1.16; *p* = 0.11) with very low CoE when looking at the best-case scenario for patch angioplasty. In the worst-case scenario, no significant difference was seen (RR 1.35; 95% CI 0.55 to 3.30; *p* = 0.51). Of the eight patients who died in the Eikelboom trial [64], it is unknown in which group they were randomized. The trial of Mannheim [54] described 79 deaths, also unknown in which group. The Kaplan-Meier figure showed similar death/survival rates, the assumption can be made that the events are spread fifty-fifty in each group. Sensitivity analysis did not show a different result compared with the best-case and worst-case scenario that was made. The trial of AbuRahma [60] mentioned that some of the patients died after 30 days, but they did not described how many.

#### Serious adverse events (SAE)

**Fig. 8:**
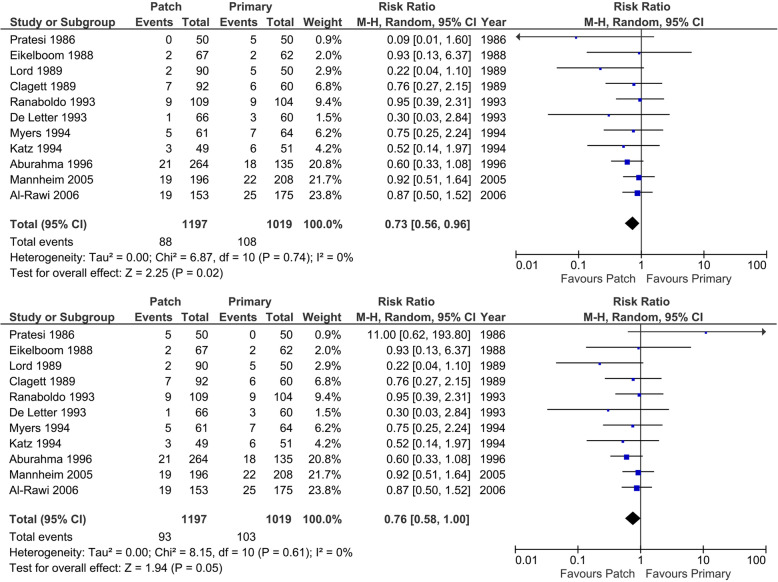
Forest plot on serious adverse events (random-effects model). **a** Best-case scenario patch angioplasty. **b** Worst-case scenario patch angioplasty

Eleven trials reported serious adverse events after surgery. In 1197 operations were 88 (best-case scenario patch) or 93 patients (worst-case scenario patch) SAE reported (7.4 to 7.8%) in the patch angioplasty group versus 103 patients (worst-case scenario patch) or 108 patients (best-case scenario patch) with SAE (10.1 to 10.6%) in 1019 operations in the primary closure group (Fig. 8). In meta-analysis, low heterogeneity was present (*I*^2^ 0%; *p* = 0.61 and 0.74), and the random-effects model showed statistically significant differences between the patch angioplasty and primary closure group (RR 0.73; 95% CI 0.56 to 0.96; *p* = 0.02) at very low CoE when looking at the best-case scenario for patch angioplasty. In the worst-case scenario, also a significant difference was seen (RR 0.76; 95% CI 0.58 to 1.00; *p* = 0.05). The trial of Pratesi [63] described 5 patients who died, but they did not describe in which group (patch or primary closure). A best-case and worst-case scenario for patch angioplasty was made.

#### Health-related quality of life

No meta-analysis of *health-related quality of life* was performed as none of the trials described health-related quality of life aspects. None of the trials reported health status.

### Secondary outcomes

#### Symptomatic or asymptomatic arterial occlusion or restenosis (50 to 99%)

**Fig. 9:**
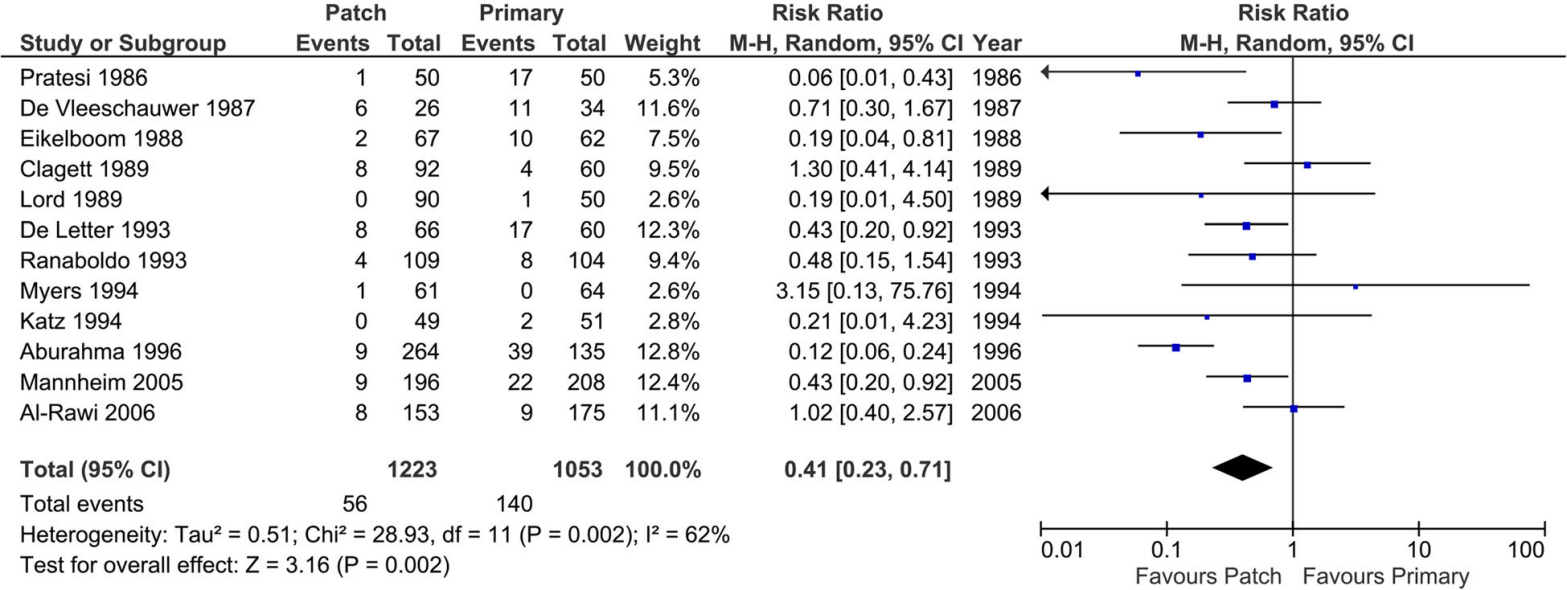
Forest plot on symptomatic or asymptomatic arterial occlusion or restenosis (50 to 99%) (random-effects model)

All trials described symptomatic or asymptomatic arterial occlusion or restenosis (50 to 99%). In the patch angioplasty group, 56 patients (3.8%) of the 1223 operations in the primary closure group versus 140 patients (11.6%) of the 1053 operations suffered from symptomatic or asymptomatic arterial occlusion or restenosis (50 to 99%) (Fig. 9).

Substantial heterogeneity was present (*I*^2^ 62%; *p* < 0.05), the random-effects model showed statistically significant differences between the patch angioplasty versus the primary closure group (RR 0.41; 95% CI 0.23 to 0.71; *p* = < 0.01) with very low CoE.

### Exploratory outcomes

#### Separately reported serious adverse events

In one trial, one patient suffered one or more SAE within 30 days after surgery. Katz [52] describes a patient, 4 weeks after initial surgery with a draining sinus tract infection after polytetrafluoroethylene (PTFE) patch placement. It was necessary to remove the PTFE patch and replace it with a venous patch.

#### Separately reported non-serious adverse events

None of the trials reported such events within 30 days after surgery.

#### Stroke < 30 days after surgery

**Fig. 10:**
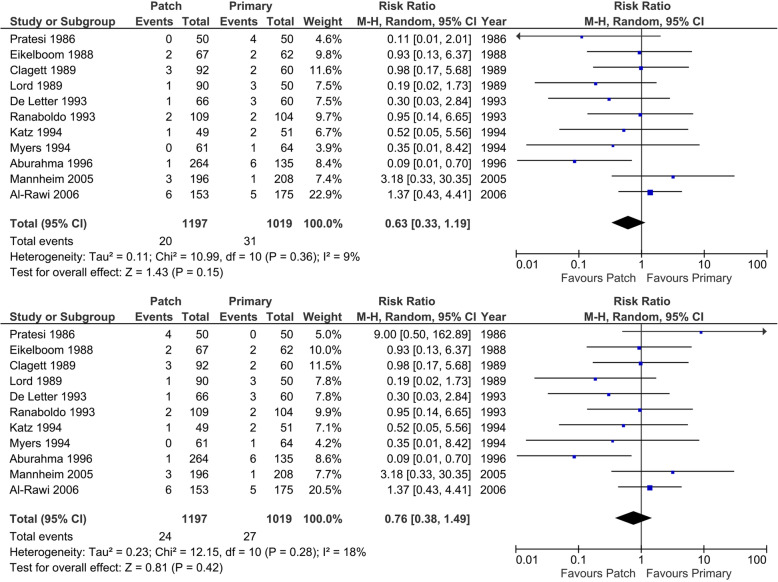
Forest plot on < 30 days stroke (random-effects model). **a** Forest plot on < 30 days stroke. Best-case scenario patch angioplasty. **b** Forest plot on < 30 days stroke. Worst-case scenario patch angioplasty

Eleven trials reported on stroke < 30 days after surgery (Fig. 10). There are a total of 20 strokes (best-case scenario patch) or 24 (worst-case scenario patch) (1.7 to 2.0%) in 1197 operations in the patch angioplasty group versus 27 (worst-case scenario patch) or 31 strokes (best-case scenario patch) (2.6 to 3.0%) in 1019 operations in the primary closure group. In meta-analysis, low heterogeneity was present (*I*^2^ 9% and 18%; *p* = 0.36 and* p* = 0.28), and the random-effects model showed no statistically significant differences between the patch angioplasty and primary closure group (RR 0.63; 95% CI 0.33 to 1.19; *p* = 0.15) with very low CoE when looking at the best-case scenario for patch angioplasty. In the worst-case scenario, no significant difference was seen (RR 0.76; 95% CI 0.38 to 1.49; *p* = 0.42). The trial of Pratesi [63] described four patients who suffered a stroke, but they did not describe in which group. A best-case and worst-case scenario for patch angioplasty was made.

*Funnel plots* were not established as funnel plot asymmetry should be used only when there are at least 10 studies included in the meta-analysis; also, the included data was considered outdated [30].

### Trial sequential analysis (TSA)

*For all outcomes*, model variance-based heterogeneity was estimated 0%. We did not perform the TSA with 25% diversity (as stated in protocol [32]) since this would only further increase the required information size.

When calculating the TSA scenarios for point-estimates obtained in the meta-analysis, we found for all-cause mortality, mortality > 30 days, SAE, and < 30 days stroke and restenosis the TSA all gave inconclusive results, underlining the information size is extremely short of the required. With mortality < 30 days, the alpha spending boundary is ignored due to too small information.

We did not compare the imprecision assessed according to GRADE with that of TSA as planned because we could not perform the primary TSAs as planned in the protocol due to too little statistical information size. Accordingly, we would have downgraded imprecision two levels with TSA, and this was already at the lowest level.

### Subgroup analysis

None of the trials showed low risk of bias and the subgroups describing different patch materials were too small, precluding the pre-planned subgroup analyses. This was due to lack of data despite several attempts at contacting the corresponding authors of the trials. None of the trials reported statin use, body mass index, or American Society of Anaesthesiologists classification reported.

## Discussion

This systematic review with meta-analysis and trial sequential analysis (TSA) included 12 RCTs. Overall, the trials randomized 2187 patients and performed 2335 carotid endarterectomies, comparing patch angioplasty versus primary closure in carotid surgery for symptomatic patients.

In this review, the percentage of death < 30 days after surgery is comparable with the highest reported in other literature [15]. In < 30 days mortality and serious adverse events (SAE), *I*^2^ was 0%; nevertheless, clinical heterogeneity is not excluded by an *I*^2^ of 0%. An explanation could be a low number of included RCTs.

The current guidelines favor patch angioplasty above primary closure after carotid surgery [8, 16, 17]. The recommendation is based on trials that did not report clearly on patient-level data (one patient with one or more SAE); instead, the trials reported a number of complications and a number of operations. We tried contacting the corresponding author of each trial at least twice, with no response at all. The lack of individual patient data prohibited us doing the statistical analysis as planned in the protocol. We evaluated the included trials according to the three dimensions of risk of error: bias, “play of chance,” and design [31]. Trials fall short on the bias protection, the included numbers of patients, and the chosen outcomes. All trials were classified at high risk of bias, as they all scored unclear or high risk of bias in one or more of the seven bias risk components (Fig. 4). For example, none of the trials conducted blinded outcomes assessment which is known to affect outcomes such as mortality [66]. Therefore, the meta-analytic effect estimates in our analyses may eventually appear to overestimate the effect when trials at low risk of bias emerge, all the included RCTs were at evidence level 1d [31].

A firm conclusion on which technique is superior cannot be drawn yet based on the current available literature. And even though firm evidence is missing, our guidelines are based on these old and uncertain trial data. We may agree with the guidelines advice to use patch angioplasty awaiting firm evidence to support this. Despite the current guideline, a small majority of the vascular surgeons use patch angioplasty when performing carotid endarterectomy. For example in the CREST-1 trial (level of evidence 1b [31]), the actual rate of patch closure was only in 62.4% of the patients [67]. Another recent review suggested a higher rate of restenosis after primary closure. The methodological quality of the included studies was considered moderate to poor [33]. The lack of current evidence at low risk of bias underscores the need for new RCTs because the (medical) treatment of patients with a carotid stenosis has been improved over the last two decades.

All included RCTs in this review are 15 to 35 years old and many aspects on handling and treating these patients changed during the last couple of years. For example, most centers are now using ultrasound to locate the lesion, and centralization of treatment has taken place. The prognosis of patients may also be influenced by several other confounding factors and/or different use of co-interventions. For example, secondary prevention has evolved during the last decades [68–71]. These outdated RCTs are more bias-prone, because randomization was less well implemented or described 20 years ago, and less applicable to current practice of vascular surgery (indirectness). Nowadays, cardiovascular risk management is well known and discussed with vascular internal specialists and general practitioners.

The included trials did not describe the following: any learning curve effect, single surgeon performance [50, 61], single surgeon trials, dedicated carotid vascular teams, large volume centers, (non-)training centers, all influencing heterogeneity. Therefore, common clinical practice and the number of patients with complication ought to be followed up through clinical databases and compared with benchmark values [30].

In the included RCTs in the current review, the available information about the degree of stenosis is scarce. The degree of stenosis and its measurements is still debated [8]. The guidelines agree to perform surgery when a symptomatic patient has a 70 to 99% stenosis. Between 50 and 69% stenosis, there is room for discussion with the patient and the caregiver [6–8]. In essence, this means that every patient with a symptomatic stenosis of > 50% will be a candidate for surgery. After extensive discussions with all co-authors and external experts, every patient with a symptomatic stenosis of the internal carotid artery > 50% was considered a candidate for surgery, but due to lack of data concerning the degree of stenosis of each patient, and the relatively small numbers of patients, (a)symptomatic patients in contrast to only symptomatic patients as stated in the protocol were included [32].

In this review, the outcome measures were graded from the patients’ point of view according to GRADE, focusing on the patient-important outcomes critical for decision making as stated in the protocol [32, 37, 72]. Overall mortality, SAE (including stroke), and restenosis were considered as such critical outcomes [32]. Future trials and studies should be thoroughly discussed, well-organized, registered, and should be protocol based before launched [73]. However, even though databases may provide large numbers of patients, and given they inform on consecutive cohorts of patients and may provide some answers of the actual status on benefits and harms, they will always be prone to the huge risk of bias introduced by the choice of intervention by indication. None of the trials included in this review are large trials (> randomized 2000 patients) in the sense that they statistically have the power to detect or exclude even rather large intervention effects on important outcomes. Therefore, future studies should plan to check their position along the three dimensions of possible errors: bias, “the play of chance,” and the choice of outcomes. It has been proven extensively that trials at low risk of bias produce more reliable results compared with trials at high risk of bias [30, 72]. Based on the above considerations, we propose to conduct new large trials at low risk of bias and using outcomes critical for decision making. These future trials should focus on comparing patch closure versus primary closure [60].

Multiple reviews have been published on this topic with similar conclusions [33, 34]. These reviews concluded that all the evidence available is of moderate quality, and future RCTs are needed. In the referred reviews, patch angioplasty seems to be favorable in terms of perioperative stroke, and restenosis (> 50% or occlusion) compared with primary closure. These reviews could have graded the available evidence down to the lowest level of certainty due to the risk of bias and imprecision. Although patch angioplasty seems favorable for certain outcomes, each review has different outcomes on which this conclusion is made. So all the available reviewed evidence is of poor quality and leads to statements and conclusions that are likely unwarranted or at least questionable. More trials at low risk of bias are needed before firm conclusions can be drawn and recommendations can be made. This important point was recently highlighted during a large global Vascular Congress (carotid sessions at VEITH) November 2019, New York, USA. A potential obstacle is the size of such RCTs, to demonstrate a difference in mortality, stroke, and internal carotid artery restenosis or occlusion [61]. The number of patients needed would be high. In this review, the sum of patients included was too small to perform TSA. TSA could have shown how many more randomized patients may be needed to draw firm conclusions. Right now the required number of patients needed to identify an effect is large [74]. Based on the performed meta-analysis, we conclude that more evidence is needed on this topic.

## Conclusions

This systematic review showed no conclusive evidence of a difference between carotid endarterectomy with patch angioplasty versus primary closure of the arterial wall on all-cause mortality, < 30 days mortality, < 30 days stroke, or any other serious adverse events. These conclusions are based on data from 15 to 35 years ago, obtained in trials with very low certainty according to GRADE, and should be interpreted cautiously. Therefore, we suggest conducting new randomized clinical trials patch angioplasty versus primary closure in carotid endarterectomy in symptomatic patients with an internal carotid artery stenosis of 50% or more. Such trials ought to be designed according to the Standard Protocol Items: Recommendations for Interventional Trials statement [75] and reported according to the Consolidated Standards of Reporting Trials statement [76]. Until conclusive evidence is obtained, the standard of care according to guidelines should not be abandoned.

## Supplementary Information

**Additional file 1.** Search strategy.

**Additional file 2.** GRADE summary of findings table.

**Additional file 3: Table S1.** (uploaded as supplementary file because of the table size).

**Additional file 4.** Review Protocol.

## Data Availability

The search strategy that was followed in the different online libraries, PubMed/Medline, The Cochrane Library, and Embase is added as supplementary file 1. The full key terms and MeSH terms are described. The protocol is added as supplementary file 4. Upon request, more data are available.

## Abbreviations

CEA: Carotid endarterectomy
CI: Confidence interval
CoE: Certainty of Evidence
ECST: European Carotid Surgery Trial
ESVS: European Society of Vascular Surgery
FWER: Family-wise error rate
GRADE: Grading of Recommendations Assessment, Development and Evaluation
ICA: Internal carotid artery
MD: Mean difference
NASCET: North American Symptomatic Carotid Endarterectomy Trial
NVvV: Nederlandse Vereniging voor Vaatchirurgie (Dutch Society of Vascular Surgery)
PRISMA: Preferred Reporting Items for Systematic reviews and Meta-Analysis
PTFE: Polytetrafluoroethylene
RCT: Randomized clinical trial
RR: Relative risk
SAE: Serious adverse events
TCD: Transcranial Doppler
TSA: Trial sequential analysis
