# Supplementary material for: Carotid endarterectomy with patch angioplasty versus primary closure in patients with symptomatic and significant stenosis: a systematic review with meta-analyses and trial sequential analysis of randomized clinical trials
Source: Syst Rev. 2021 May 6;10:139. doi: 10.1186/s13643-021-01692-8 (PMC8103619; doi:10.1186/s13643-021-01692-8)
Supplement: Supplementary file 1 — Additional file 1. Search strategy. [file 13643_2021_1692_MOESM1_ESM.pdf]

Supplementary file I Searches

Primary search.

Search PubMed 13th June 2019

(("Endarterectomy, Carotid"[Mesh] OR "Carotid Stenosis"[Mesh] OR "Stents"[Mesh] OR (Carotid[tiab] AND Endarterectomy[tiab]) OR (eversion[tiab] AND endarterectomy[tiab) OR (eversion[tiab] AND CEA[tiab]) OR eCEA[tiab] OR Carotid Stenos*[tiab] OR (carotid[tiab] AND Stent*[tiab]) OR (carotid[tiab] AND surger*[tiab])) AND ("Blood Vessel Prosthesis"[Mesh] OR "Polyethylene Terephthalates"[Mesh] OR "Polytetrafluoroethylene"[Mesh] OR Polytef[tiab] OR PTFE[tiab] OR TFE[tiab] OR FEP[tiab] OR Tarflen[tiab] OR Fluoroplast[tiab] OR GORE-TEX[tiab] OR Goretex[tiab] OR Teflon[tiab] OR Fluon[tiab] OR Polyethylene Terephthalate[tiab] OR Dacron[tiab] OR Polytetrafluoroethylene[tiab] OR Patch*[tiab] OR Blood Vessel Prosthes*[tiab] OR Vascular Prosthes*[tiab] OR Tissue-Engineered Vascular Graft*[tiab] OR "Angioplasty"[Mesh] OR angioplast*[tiab] OR biopatch*[tiab] OR porcine[tiab] OR bovine[tiab] OR "Endarterectomy, Carotid"[Mesh] OR (carotid[tiab] AND endarterectomy[tiab]) OR cCEA[tiab]))

42462 hits

Search EMBASE 13th June 2019

(('carotid endarterectomy'/exp OR 'carotid artery obstruction'/exp OR 'carotid artery stenting'/exp OR 'carotid artery stent'/exp or (Carotid:ti,ab AND Endarterectomy:ti,ab) OR (eversion:ti,ab AND endarterectomy:ti,ab) OR (eversion:ti,ab AND CEA:ti,ab) OR ‘Carotid Stenos*’:ti,ab OR (carotid:ti,ab AND Stent*:ti,ab) OR (carotid:ti,ab AND surger*:ti,ab)) AND ('blood vessel prosthesis'/exp OR 'polyethylene terephthalate'/exp OR 'polytetrafluoroethylene covered stent'/exp OR Polytef:ti,ab OR PTFE:ti,ab OR TFE:ti,ab OR FEP:ti,ab OR Tarflen:ti,ab OR Fluoroplast:ti,ab OR ‘GORE-TEX’:ti,ab OR Goretex:ti,ab OR Teflon:ti,ab OR Fluon:ti,ab OR ‘Polyethylene Terephthalate’:ti,ab OR Dacron:ti,ab OR Polytetrafluoroethylene:ti,ab OR Patch*:ti,ab OR ‘Blood Vessel Prosthes*’:ti,ab OR ‘Vascular Prosthes*’:ti,ab OR ‘Tissue-Engineered Vascular Graft*’:ti,ab OR 'angioplasty'/exp OR angioplast*:ti,ab OR biopatch*:ti,ab OR porcine:ti,ab OR bovine:ti,ab OR 'carotid endarterectomy'/exp OR (carotid:ti,ab AND endarterectomy:ti,ab) OR cCEA:ti,ab))

25898 hits

Search Cochrane 13th June 2019

Carotid Endarterectomy OR eversion endarterectomy OR eversion CEA OR eCEA OR Carotid Stenos* OR carotid Stent* OR carotid surger*

AND

Polytef OR PTFE OR TFE OR FEP OR Tarflen OR Fluoroplast OR GORE-TEX OR Goretex OR Teflon OR Fluon OR Polyethylene Terephthalate OR Dacron OR Polytetrafluoroethylene OR Patch* OR Blood Vessel Prosthes* OR Vascular Prosthes* OR Tissue-Engineered Vascular Graft* OR angioplast* OR biopatch* OR porcine OR bovine OR carotid endarterectomy OR cCEA

1453 hits

Search Google Scholar 13th June 2019

Carotid Endarterectomy in title

1000 hits

Updated search.

The primary search mentioned above was updated after expert suggestion on the 1st of June 2020 including the new outcome. There were not found any randomized clinical trials comparing patch versus primary closure in carotid endarterectomy.
