# Supplementary material for: Carotid endarterectomy with patch angioplasty versus primary closure in patients with symptomatic and significant stenosis: a systematic review with meta-analyses and trial sequential analysis of randomized clinical trials
Source: Syst Rev. 2021 May 6;10:139. doi: 10.1186/s13643-021-01692-8 (PMC8103619; doi:10.1186/s13643-021-01692-8)
Supplement: Supplementary file 2 — Additional file 2. GRADE summary of findings table. [file 13643_2021_1692_MOESM2_ESM.zip › GRADE SoF table (best case scenario patch) 22032021R2.docx]

Patch angioplasty compared to primary closure for patients with a significant and symptomatic stenosis (best case scenario patch)

| **Certainty assessment** | | | | | | | **№ of patients** | | **Effect** | | **Certainty** | **Importance** |
| --- | --- | --- | --- | --- | --- | --- | --- | --- | --- | --- | --- | --- |
| **№ of studies** | **Study design** | **Risk of bias** | **Inconsistency** | **Indirectness** | **Imprecision** | **Other considerations** | **patch angioplasty** | **primary closure** | **Relative (95% CI)** | **Absolute (95% CI)** |  |  |
| **all-cause mortality at maximum follow up (follow up: range 1 month to 96 months)** | | | | | | | | | | | | |
| 10 | randomized trials | serious ^a^ | serious ^b^ | serious ^c^ | serious ^d^ | none | 82/1145 (7.2%) | 171/969 (17.6%) | **RR 0.53** (0.26 to 1.08) | **83 fewer per 1.000** (from 131 fewer to 14 more) | ⨁◯◯◯ VERY LOW |  |
| **< 30 days mortality (follow up: range 1 month to 96 months)** | | | | | | | | | | | | |
| 10 | randomized trials | serious ^a^ | not serious ^b^ | serious ^c^ | very serious ^e^ | none | 6/1107 (0.5%) | 8/969 (0.8%) | **RR 0.61** (0.21 to 1.71) | **3 fewer per 1.000** (from 7 fewer to 6 more) | ⨁◯◯◯ VERY LOW |  |
| **>30 days mortality (follow up: range 1 month to 96 months)** | | | | | | | | | | | | |
| 9 | randomized trials | serious ^a^ | serious ^b^ | serious ^c^ | serious ^d^ | none | 65/1057 (6.1%) | 163/919 (17.7%) | **RR 0.52** (0.23 to 1.16) | **85 fewer per 1.000** (from 137 fewer to 28 more) | ⨁◯◯◯ VERY LOW |  |
| **SAE except death (follow up: range 1 month to 96 months)** | | | | | | | | | | | | |
| 11 | randomized trials | serious ^a^ | not serious ^b^ | serious ^c^ | serious ^d^ | none | 88/1197 (7.4%) | 108/1019 (10.6%) | **RR 0.73** (0.56 to 0.96) | **29 fewer per 1.000** (from 47 fewer to 4 fewer) | ⨁◯◯◯ VERY LOW |  |
| **Symptomatic or asymptomatic restenosis (50-99%) or occlusion (follow up: range 1 month to 96 months)** | | | | | | | | | | | | |
| 12 | randomized trials | serious ^a^ | serious ^b^ | serious ^c^ | serious ^d^ | none | 56/1223 (4.6%) | 140/1053 (13.3%) | **RR 0.41** (0.23 to 0.71) | **78 fewer per 1.000** (from 102 fewer to 39 fewer) | ⨁◯◯◯ VERY LOW |  |
| **< 30 days stroke (follow up: range 1 month to 96 months)** | | | | | | | | | | | | |
| 11 | randomized trials | serious ^a^ | not serious ^b^ | serious ^c^ | very serious ^e^ | none | 20/1197 (1.7%) | 31/1019 (3.0%) | **RR 0.63** (0.33 to 1.19) | **11 fewer per 1.000** (from 20 fewer to 6 more) | ⨁◯◯◯ VERY LOW |  |

**CI:** Confidence interval; **RR:** Risk ratio

#### Explanations

a. Downgraded by one level because of risk of bias due to unclear randomization (sequence generation and/or allocation concealment) and lack of blinding.

b. Downgraded by one level because of statistical heterogeneity of trial results (I-square greater than 50%).

c. Downgraded by one level because of indirectness caused by differences between the standard of medical care in the trials (conducted 15 to 35 years ago) and today's standard of medical care.

d. Downgraded by one level because of imprecision caused by unclearly reported events, that required best- and worst-case scenario analyses with divergent results

e. Downgraded by two levels because of imprecision caused by unclearly reported events, that required best- and worst-case scenario analyses with divergent results and also a small population with a low event rate
