# Supplementary material for: Carotid endarterectomy with patch angioplasty versus primary closure in patients with symptomatic and significant stenosis: a systematic review with meta-analyses and trial sequential analysis of randomized clinical trials
Source: Syst Rev. 2021 May 6;10:139. doi: 10.1186/s13643-021-01692-8 (PMC8103619; doi:10.1186/s13643-021-01692-8)
Supplement: Supplementary file 3 — Additional file 3: Table S1. (uploaded as supplementary file because of the table size). [file 13643_2021_1692_MOESM3_ESM.docx]

| Author and year | Total ptns | Number of procedures | | Total procedures | Age (yr) | | Sex | | | Period | Follow up | Country (m/s) | Smoking | | | | | Diabetes | | | Hypertension | | | Coronary disease | | PAD/previous vascular surgery | | |
| --- | --- | --- | --- | --- | --- | --- | --- | --- | --- | --- | --- | --- | --- | --- | --- | --- | --- | --- | --- | --- | --- | --- | --- | --- | --- | --- | --- | --- |
|  |  | Primary | Patch |  | Primary | Patch | Primary | | Patch |  |  |  | Primary | | Patch | | | Primary | | Patch | Primary | Patch | | Primary | Patch | Primary | | Patch |
| Pratesi, 1986 | 90 | 50 | 50 | 100 | 63 | | 76M  14F | | | 5 year | 48 months | Italy (s) | 74 | | | | | 17 | | | 39 | | | U | | U | | |
| De Vleeschauwer 1987 | 94 | ≥34 | ≥26 | ≥60 | 60.7 | | 62M 32F | | | 12-1984—10-1985 | 12 months | Germany (s) | U | | | | | U | | | U | | | U | | U | | |
| Eikelboom 1988 | 129 | 62 | 67 | 129 | 63 | 63 | 46M 16F | 49M 18F | | 01-1984 – 12-1985 | 12 months | Netherlands (s) | U | | | | | 3 | 9 | | 40 | | 33 | 26 | 27 | 26 | 22 | |
| Clagett,1989 | 136 | 60 | 92 | 152 | 62 | 62 | 135M  1F (patch) | | | 4 years | 22 months | USA (s) | 95% | | | 93%VP 93%OV | | 17% | 18%VP 23%OV | | 60% | | 50%VP 67%OV | U | | U | | |
| Lord 1989 | 123 | 50 | 43 V 47 PTFE | 140 | 63 | 62.9 V  65.5 PTFE | 33M 17F | | 27M/ 16F V  27M/20F PTFE | 02-1985—10-1986 | U | Australia (s) | 12C | 21*P* | 13C 12C | | 23P 20P | 4 | | 4 V  2 PTFE | 26 | 29 V 29 PTFE | | 22 | 23 V 23 PTFE | U | | U |
| Ranaboldo 1993 | 199 | 104 | 53 V 56 D | 213 | 65.9 | | 148M 65F | | | ? | 12 months | England (m) | U | | | | | U | | | U | | | U | | U | | |
| Katz 1994 | 87 | 51 | 49 | 100 | 69 | 66 | 25M 19F | | 24M 19F | 02-1988—10-1989 | 29.2 | USA (s) | 21 | | 24 | | | 7 | | 9 | 29 | 25 | | 18 | 14 | 17 | | 16 |
| DeLetter,1993 | 126 | 62 | 67 | 129 |  |  | 44M 16F | |  | 1 year | Mean 60 months (range 1-96) | Netherlands (s) | U | | | | | U | | | U | | | U | | U | | |
| Myers1994 | 136 | 64 | 61 V  38 OV | 163 | 62.1 | 61.8 V 61.4 OV | 64M  0F | | 60M 1F V  38M OV | 4 year | 57 months ±4 PC 59 months ±4.2 VP  62 months ±4.2 OVP | USA (s) | 95% | | 93% OV | | | 17% | | 18%  23% | 60% | 50% V  67% OV | | U | | U | | |
| Aburahma 1996/2004 | 315 unilateral  42 bilateral | 135 | 130 V 134 PTFE | 399 | 68.4 | 67.9 PTFE  68.0 SV  68.2 JV | 71M 64F | | 71M63F PTFE  34M36F SV  29M31F JV | 10-1991—11-1995 | Mean 30 months (range 1-62) | USA (s) | 84 | | 74 PTFE  42 SV  33 JV | | | 34 | | 36 PTFE  17 SV  12 JV | 109 | 99 PTFE  54 SV  47 JV | | 86 | 76 PTFE  35 SV  36 JV | U | | |
| Mannheim 2005 | 404 | 208 | 196 | 422 | 71 | 68 | 131M 77F | | 134M 62F | 02-1999—03-2002 | 5 years | Israel (s) | 34 | | 48 | | | 74 | | 76 | 155 | 146 | | 108 | 95 | 72 | | 71 |
| Al-Rawi 2006 | 306 | 175 | 153 | 328 | 68 | 70 | 207M 99F | | | 04-1997—01-2003 | 12 months Median 398 days | England (s) | 37 | | 30 | | | 30 | | 24 | 106 | 89 | | 57 | 49 | 35 | | 18 |

Table S1: baseline characteristics of randomized CEA patients with patch angioplasty and CEA patients with primary closure of all included trials. Author = first author of paper, year and reference, ptns= patients s = single center, m = multicenter, M = male, F = female, n = number of patients, U = unknown, C= Current smoker, P = Past smoker. PAD= peripheral arterial disease, V= vein patch, D= dacron patch, PTFE= polytetrafluoroethylene patch. OV=obligatory vein patch, SV= saphenous vein patch, JV= Jugular vein patch. PC = primary closure. The use of electroencephalography was only described in the trial of Al-Rawi.^55^ Acetylic acid was used in all trials as platelet inhibitor.
